# The echocardiographic pulmonary to left atrial ratio: A noninvasive variable for the hemodynamic classification of pulmonary hypertension in dogs

**DOI:** 10.1111/jvim.17097

**Published:** 2024-05-07

**Authors:** Andrea Corda, Francesca Corda, Plamena Pentcheva, Mariangela Puci, Alessandra Mollica, Pablo Gomez Ochoa, Thouraya Dabbagh, Maria Luisa Pinna Parpaglia

**Affiliations:** ^1^ Veterinary Teaching Hospital, Department of Veterinary Medicine University of Sassari Sassari Italy; ^2^ Clinical Epidemiology and Medical Statistic Unit, Department of Medicine, Surgery and Pharmacy University of Sassari Sassari Italy; ^3^ VetCorner Unavets Zaragoza Spain

**Keywords:** combined, dog, echocardiography, isolated, left heart disease, lungs, mitral valve disease, postcapillary, precapillary, pulmonary circulation, pulmonary hypertension, respiratory disease

## Abstract

**Background:**

Hemodynamic classification of pulmonary hypertension (PH) has important clinical implications. However, only a few echocardiographic variables have been used to hemodynamically classify PH in dogs.

**Objective:**

To evaluate the echocardiographic pulmonary to left atrial ratio index (ePLAR) in dogs with PH.

**Animals:**

Forty‐six dogs with intermediate to high probability of PH.

**Methods:**

Cross‐sectional study. Variables were compared between dogs with precapillary PH [PrePH (n = 24)] vs postcapillary PH [PostPH (n = 22)], and with combined PH [CombPH (n = 14)] vs isolated PH [IsoPH (n = 8)] using the *t*‐, Mann‐Whitney, Pearson's Chi, or Fisher's exact test. The receiver operating characteristic curve and Youden index were used to identify the optimal ePLAR cutoff value to differentiate among the groups, intraclass correlation coefficients (ICC) were used to determine the reliability of measurements.

**Results:**

The mean (SD) ePLAR of the PrePH was higher than that of the PostPH group [0.36 (0.13) vs 0.26 (0.09), respectively; *P* = .005]. The median (interquartile range) ePLAR of the CombPH was higher than that of the IsoPH subgroup [0.29 (0.24‐0.38), vs 0.20 (0.16‐0.23), respectively; *P* = .001]. The best cutoff value of ePLAR for identifying IsoPH was <0.245 [AUC at cutoff point = 0.86; sensitivity (95% confidence interval [CI]) = 0.71 (0.47‐0.95); specificity (95% CI) = 1 (0.76‐1)]. The ICC analysis indicated a high degree of reliability.

**Conclusions and Clinical Importance:**

ePLAR can be considered a valid noninvasive variable to hemodynamically classify PH in dogs with an intermediate to high probability of PH. Assessment of ePLAR can be useful in the therapeutic management of PH in dogs.

**Abbreviations**
AC: Andrea Corda
ACEi: angiotensin converting enzyme inhibitors
Ao: aorta
AT: pulmonary flow acceleration time
Bpm: beats per minute
BW: body weight
CFD: color flow Doppler
CIPF: canine idiopathic pulmonary fibrosis
CV: cardiovascular
CVC: caudal cava vein
CW: continuous wave
DCM: dilated cardiomyopathy
EF: ejection fraction
ePLAR: echocardiographic pulmonary to left atrial ratio
ET: pulmonary flow ejection time
F: furosemide
FC: Francesca Corda
FAC: fractional area change
FS: fractional shortening
HR: heart rate
HWD: heartworm disease
IQR: interquartile range
IVS: interventricular septum
LA: left atrium
LAP: left atrial pressure
LHD: left‐sided heart disease
LV: left ventricle
LVIDd: left ventricular internal diameter in diastole
LVIDdN: left ventricular internal diameter in diastole normalized for body weight
LVIDs: left ventricular internal diameter in systole
LVIDsN: left ventricular internal diameter in systole normalized for body weight
MMVD: myxomatous mitral valve disease
OAD: obstructive airway disorders
P: pimobendan
PA: main pulmonary artery
PAP: pulmonary arterial pressure
PCWP: pulmonary capillary wedge pressure
PDE5i: phosphodioesterase 5 inhibitors
PF: pulmonary flow
PH: pulmonary hypertension
PostPH: postcapillary pulmonary hypertension
PR: pulmonary regurgitation
PrePH: precapillary pulmonary hypertension
PRV: pulmonary regurgitation velocity
PT: pulmonary thrombosis
PTE: pulmonary thromboembolism
PVP: pulmonary venous pressure
PVR: pulmonary vascular resistance
PVRecho: echocardiographic‐derived pulmonary vascular resistance
PW: pulsed wave
RA: right atrium
RAA: right atrial area
RHC: right heart catheterization
RHF: right‐sided congestive heart failure
RPAD: right pulmonary artery distensibility
RV: right ventricle
RVOT FS: right ventricular outflow tract fractional shortening
S: sildenafil
TAPSE: tricuspid annular plane systolic excursion
TDI: tissue Doppler imaging
TPG: transpulmonary pressure gradient
TR: tricuspid regurgitation
TRV: tricuspid regurgitation velocity

## 1 INTRODUCTION

Pulmonary hypertension (PH) is characterized by abnormally high pulmonary arterial pressure (PAP). This condition can result from increased pulmonary blood flow, increased pulmonary vascular resistance (PVR), elevated pulmonary venous pressure (PVP), or a combination of them. Hemodynamically, PH is classified into 2 main groups, precapillary (PrePH) and postcapillary (PostPH), resulting from abnormalities on the arterial side of the pulmonary vascular system and pulmonary venous congestion, respectively. Precapillary PH occurs when there is an elevation in PAP because of increased PVR, with normal pulmonary capillary wedge pressure (PCWP).^1^, ^2^ In dogs, PrePH is caused by chronic respiratory diseases/hypoxia,^3^, ^4^, ^5^, ^6^, ^7^, ^8^, ^9^, ^10^, ^11^, ^12^, ^13^ thromboembolic disease,^14^ cardiopulmonary parasitoses,^15^, ^16^, ^17^ congenital systemic‐to‐pulmonary shunts,^8^, ^9^, ^10^, ^18^, ^19^, ^20^, ^21^, ^22^ and idiopathic pulmonary arterial hypertension.^23^ Postcapillary PH is an elevated PAP secondary to increased PVP and PCWP.^1^, ^2^ It occurs most commonly in dogs with left‐sided heart disease (LHD) that have increased left atrial pressure (LAP), such as myxomatous mitral valve (MMVD) and myocardial diseases.^2^, ^24^ Other causes of PostPH can be pulmonary veno‐occlusive disease and pulmonary capillary hemangiomatosis.^25^, ^26^, ^27^ Postcapillary PH can be further classified into isolated PH (IsoPH) and combined pre‐ and postcapillary PH (CombPH). Isolated PH is characterized by increased PAP with no secondary reactive pulmonary arterial remodeling, and thus, normal PVR. In contrast, CombPH is characterized by structural changes in the pulmonary vasculature caused by chronic and progressive congestion of the pulmonary capillaries and veins, which increase PVR.^24^, ^28^ The gold standard method for the diagnosis and classification of PH is the direct assessment of PAP and PCWP using right heart catheterization (RHC).^1^ However, performing RHC is impractical because of the invasiveness of the procedure and the anesthetic risk in cases of severe cardiorespiratory disorders. In dogs, the echocardiographic distinction between PrePH and PostPH is based on the left atrial (LA) dimension,^2^, ^13^, ^29^, ^30^, ^31^, ^32^, ^33^ and the differentiation between IsoPH and CombPH is based on the severity of PH in subjects with LHD and LA enlargement.^2^, ^34^ The correct hemodynamic classification of PH is of fundamental importance to optimize therapeutic management.^2^, ^35^, ^36^ In fact, pulmonary artery vasodilators, such as phosphodioesterase 5 inhibitors (PDE5i), could be useless or even contraindicated in patients with PostPH as they could potentially exacerbate pulmonary edema.^2^, ^36^, ^37^ Therefore, noninvasive echocardiographic indices that are useful for differentiating these pathophysiological conditions in clinical settings must be identified.

The echocardiographic pulmonary to left atrial ratio index (ePLAR) is useful in differentiating PrePH from PostPH in dogs^38^ and also IsoPH from CombPH in humans.^39^ The ePLAR index is obtained from the ratio between tricuspid regurgitation velocity (TRV) and the quotient of the transmitral pulsed‐wave (PW) Doppler‐derived early diastolic wave velocity (*E*) and the mitral annular septal Tissue Doppler Imaging (TDI)‐derived early diastolic wave velocity (*E*′), as follows^39^:

$$\text{EPLAR}\;\left(m/s\right)=\frac{\mathrm{TRV}\;\left(m/s\right)}{\text{septal}\;E/E^{\prime }},$$

where TRV represents a surrogate of PAP,^2^ and septal *E*/*E*′ represents an estimate of the LAP and PCWP.^40^, ^41^, ^42^

The present study aimed to evaluate the ability of ePLAR to differentiate PrePH from PostPH and IsoPH from CombPH in dogs with PH. We hypothesized that ePLAR might provide additional information for the hemodynamic classification of PH in dogs.

## 2 MATERIALS AND METHODS

This observational cross‐sectional study was conducted between March 2022 and July 2023 at the Veterinary Teaching Hospital, University of Sassari, Italy. The study protocol was reviewed and approved by the Ethical Committee for Animal Welfare of the University of Sassari (OPBSA) with protocol number 32346 (March 18, 2022). All dog owners signed an informed consent form prior to enrollment.

### 2.1 Animals

All dogs included in this study were referred for cardiac or respiratory problems. Signalment [breed, sex, age, body weight (BW)], clinical history, physical examination findings, laboratory test results, and medications, if administered, were recorded. All dogs underwent thoracic radiography and echocardiography with simultaneous ECG. Blood pressure was measured using oscillometric method (Memodiagnostic MD 15/90 Pro, S + B medVET, Babenhausen, Germany), as previously described.^43^ Other diagnostic tests such as abdominal and/or thoracic ultrasound, bronchoscopy, and laboratory test [including CBC (Lasercyte, Idexx Laboratories, Westbrook, Maine, USA), biochemical profile (ABX Pentra 400, Horiba Medical, Kyoto, Japan), urinalysis, heartworm (SNAP Heartworm test, Idexx Laboratories, Westbrook, Maine, USA, and Knott test), and angiostrongylosis tests (IDEXX Angio detect, Idexx Laboratories, Westbrook, Maine, USA, and Baermann test)] were performed, when necessary, to confirm a diagnosis. We excluded dogs with obstruction of the right ventricular outflow tract, atrial fibrillation, or other persistent nonsinus arrhythmias and dogs of breeds typically predisposed to arrhythmogenic right ventricular cardiomyopathy (such as boxers and bulldogs). Moreover, we excluded dogs with systolic arterial pressure outside the reference range (120‐160 mm Hg)^43^, ^44^ and subjects with concurrent diseases potentially associated with both PrePH and PostPH (such as, LHD with left atrial to aorta ratio [LA/Ao] ≥1.6 with concurrent respiratory, heartworm, neoplastic, and endocrine diseases). Only dogs with a measurable TRV and PH, defined as having an intermediate or high probability of PH, were included in this study. According to the ACVIM guidelines,^2^ the probability of PH was established based on TRV and the presence of echocardiographic changes suggestive of PH in (1) ventricles, (2) pulmonary artery, and (3) right atrium/caudal vena cava (Table 1).

**TABLE 1:** Anatomical sites of echocardiographic signs of PH used to assess PH probability in dogs.

Anatomical sites^2^		
Ventricles	Pulmonary artery	Right atrium/caudal vena cava
Flatting of the IVS^52^	PA/Ao >1^2^	RAA index >12.3 cm^2^/m^2^ ^59^
RV hypertrophy or dilatation or both^53^	PRV >2.5 m/s^2^	CVC enlargement^60^
TAPSE/Ao or TAPSE/Ao adjusted ≤0.65^56^	RPAD <30%^2^	
RV FAC lower than reference intervals^55^	AT/ET <0.3^75^	
RVOT FS <45%^57^	Notching of the PA flow profile^53^	

Abbreviations: AT/ET, pulmonary flow acceleration time to ejection time ratio; CVC, caudal vena cava; IVS, interventricular septum; PA, main pulmonary artery; PA/Ao, pulmonary artery to aortic diameter ratio; PRV, pulmonary regurgitation velocity; RAA, right atrial area; RPAD, right pulmonary artery distensibility index; RV FAC, right ventricular fractional area change; RV, right ventricle; RVOT FS, right ventricular outflow tract fractional shortening; TAPSE/Ao, tricuspid annular plane systolic excursion normalized to aortic diameter.

Specifically, we defined the following as having an intermediate probability of PH:

1. Dogs with TRV ≤3 m/s showing echocardiographic signs of PH in 2 of the 3 anatomical sites listed in Table 1;
2. Dogs with TRV >3 m/s without any echocardiographic signs of PH at the 3 anatomical sites listed in Table 1;
3. Dogs with TRV >3 to ≤3.4 m/s showing echocardiographic signs of PH at only 1 of the 3 anatomical sites listed in Table 1.

We defined the following as having a high probability of PH:

1. Dogs with TRV ≤3 m/s with echocardiographic signs of PH in all 3 anatomical sites listed in Table 1;
2. Dogs with TRV >3 to ≤3.4 m/s with echocardiographic signs of PH in at least 2 of the 3 anatomical sites listed in Table 1;
3. Dogs with TRV >3.4 m/s with echocardiographic signs of PH in at least 1 of the 3 anatomical sites listed in Table 1.

Dogs with PH were then classified in the following groups:

*PrePH group*: included dogs with PH of precapillary origin; they had intermediate or high probability of having PH, without echocardiographic signs of LHD, with the exception of dogs with mild MMVD, defined as the presence of regurgitant jet area/LA area <30%,^45^ and LA/Ao of <1.6.
*PostPH group*: included dogs with PH of postcapillary origin; they had an intermediate to high probability of having PH, with echocardiographic signs of LHD, and LA/Ao of ≥1.6.

Dogs of the PostPH group were further classified in the following subgroups:

*IsoPH subgroup*: included dogs with presumed isolated postcapillary PH, defined as dogs with LHD, LA/Ao of ≥1.6, an intermediate to high probability of having PH, and echocardiography‐derived pulmonary vascular resistance (PVRecho) of <1.11.
*CombPH subgroup*: included dogs with presumed combined pre‐ and postcapillary PH, defined as dogs with LHD, LA/Ao ≥1.6, a high probability of having PH, and PVRecho of ≥1.11.

Dogs were diagnosed with right heart failure (RHF) if they presented with radiographic and ultrasonographic evidence of body cavity transudative effusion without any abnormality other than PH that might be responsible.

### 2.2 Echocardiography

Echocardiography was performed by a single experienced operator [Andrea Corda (AC)] with a portable ultrasound unit (My Lab Alpha, Esaote, Florence, Italy) equipped with a multifrequency (1‐4 MHz) phased array transducer (SP2430), according to the recommendations for standard transthoracic echocardiography in dogs.^46^ During examinations, nonsedated dogs were gently restrained in the right and left lateral recumbency on an echocardiography table.

M‐mode images of the left ventricle (LV) were obtained from the right parasternal short‐axis view at the level of the papillary muscles to assess the LV internal diameters in diastole (LVIDd) and systole (LVIDs), and LV fractional shortening (FS). The LVIDd and LVIDs were normalized to BW, as reported by Cornell et al.^47^ Left ventricular ejection fraction (EF) was calculated using the biplane Simpson's method of disks from the right parasternal long‐axis 4‐chambers view, and the left apical 4‐chambers view.^48^ The LA and Ao internal diameters were measured from the right parasternal short‐axis view at the level of the heart base. The short‐axis diameter of the Ao was measured along the commissure between the noncoronary and right coronary valve cusps at early ventricular diastole in the first frame after Ao valve closure. The LA was measured in the same frame using a line extending from and parallel to the commissure between the noncoronary and left coronary Ao valve cusps up the opposite edge of the LA, without including the pulmonary veins.^49^

The mitral valve was visually assessed using B‐mode echocardiography and color flow Doppler (CFD) from both right parasternal and left apical views. MMVD was diagnosed by the presence of mitral regurgitation on CFD imaging in addition to thickened leaflets and/or mitral valve prolapse.^50^ Dogs with MMVD were classified according the ACVIM guidelines.^51^

Flattening of the interventricular septum (IVS), right ventricular (RV) hypertrophy, and dilatation were evaluated from the right parasternal short and long axis views.^52^, ^53^ Tricuspid annular plane systolic excursion (TAPSE) and RV fractional area change (FAC) were obtained from the left apical 4‐chamber view, optimized for the right heart, with M‐ and B‐mode echocardiography, respectively.^54^, ^55^ The TAPSE/Ao ratio was used as an index of RV systolic function.^56^ Right ventricular outflow tract fractional shortening (RVOT FS) was obtained by B‐ and M‐mode recordings of the RVOT, from the right parasternal short axis view.^57^ The right pulmonary artery distensibility index (RPAD) was obtained from the M‐mode scan of the right pulmonary artery, obtained from a modified right parasternal long axis view.^15^, ^58^ The main pulmonary artery (PA) to Ao ratio (PA/Ao), as well as the Doppler‐derived measurements of the pulmonary arterial flow (PAF), and regurgitation (when present) were obtained from the right parasternal short axis view at the level of heart base.^2^, ^53^ Right atrial area (RAA) and caudal cava vein (CVC) dimensions were evaluated from the left apical long axis views optimized for the right heart.^59^, ^60^

TR was evaluated by inspecting the tricuspid valve from multiple views using CFD, and the TRV was obtained using the continuous‐wave (CW) Doppler method under CFD guidance, and measured as recommended.^2^

Spectral PW transmitral flow velocities (E and A waves) and TDI of the septal mitral annulus velocities (*E*′ and *A*′ waves) were obtained from the left apical 4‐chamber view.^42^ The PVRecho was calculated using the TRV and velocity‐time integral of the pulmonary artery flow^61^ (PAF VTI), as follows:

$$\text{PVRecho}=\frac{\mathrm{TRV}\;{\left(m/s\right)}^{2}}{\mathrm{PAF}\;\mathrm{VTI}\;\left(\mathrm{cm}\right)}.$$

All echocardiographic images and loops were stored and analyzed offline by AC or by Francesca Corda (FC) under direct supervision of AC; for each echocardiographic variable, 3 to 5 consecutive measurements were averaged, and the mean values were used for further statistical analyses.

The ePLAR index was calculated from the ratio between TRV (m/s) and the quotient of the *E*‐wave velocity divided by the septal *E*′‐wave velocity (*E*/*E*′)^39^:

$$\text{ePLAR}\;\left(m/s\right)=\frac{\mathrm{TRV}\;\left(m/s\right)}{E/E^{'}\;\text{sept}.}.$$

To determine the intraobserver ePLAR measurement reliability, 10 echocardiograms from 10 different dogs were randomly selected and subjected to 2 repeated measurements by the same observer (AC) on 2 different days, 1 week apart. The same echocardiograms were measured by a second operator (FC) to assess the interoperator ePLAR measurement reproducibility.

### 2.3 Statistical analysis

Quantitative variables were described as mean and standard deviation (SD) or median and interquartile range (IQR) according to the distribution. Qualitative variables were described as absolute and relative frequencies (percentages). Differences between groups were evaluated using unpaired *t*‐ or Mann‐Whitney *U* tests (for quantitative variables) and Pearson Chi or Fisher exact tests (for qualitative variables). Intraclass correlation coefficients (ICC) were used to determine the reliability of ePLAR measurements.^62^ The receiver operating characteristic (ROC) curve and Youden index were used to identify the optimal ePLAR cutoff value to differentiate the study groups.

Statistical significance was defined as *P* < .05; statistical analyses were performed using STATA17 software (StataCorp, College Station, Texas, USA).

## 3 RESULTS

During the study period, 46 dogs with measurable TRV and an intermediate to high probability of PH were included. In all dogs, TRV was measured from the left apical view optimized for the right heart because we obtained the best alignment between the direction of the TR jet and the CW Doppler cursor from this view.

The dogs included 32 (70%) males (6 were neutered) and 14 (30%) females (8 were spayed). Twenty‐three (50%) were mongrel dogs, 4 (8.7%) dachshunds, 4 (8.7%) Chihuahua, 3 (6.5%) CKCS, 2 (4.3%) Jack Russel terriers, 2 (4.3%) Labrador retrievers, and 1 (2.2%) of each of the following breeds: Maltese, West Highland White Terrier (WHWT), Yorkshire Terrier, Springer Spaniel, Shih‐tzu, American Staffordshire Terrier, Beagle, and Great Dane.

Twenty‐four dogs (52%) were included in the PrePH group and 22 (48%) were included in the PostPH group. Of the latter, 14 dogs (64%) were affected by CombPH (CombPH subgroup) and 8 dogs (36%) were affected by IsoPH (IsoPH subgroup). A comparison of demographic variables between the study groups and subgroups is shown in Table 2. The BW was significantly lower in the PostPH group than in the PrePH group. Pulmonary hypertension probability, underlying diseases, reported clinical signs, radiographic evidence of pulmonary edema, clinical evidence of RHF, and cardiovascular medications administered to the dogs at the time of inclusion in the study are reported in Table 3.

**TABLE 2:** Comparison of age, BW, sex, HR, between PrePH and PostPH groups and between CombPH and IsoPH subgroups.

Variables	PrePH (n = 24)	PostPH (n = 22)	*P*‐value
Age, years, median (IQR)	12 (9.5‐13)	12 (10‐14)	.51
BW, kg, median (IQR)	13.3 (9.9‐20.5)	8.5 (5‐15)	.03*
Sex, male, n (%)	18 (75)	14 (64)	.40

Variables	CombPH (n = 14)	IsoPH (n = 8)	*P*‐value
Age, years, median (IQR)	12 (10‐14)	11.5 (10.5‐14.0)	.86
BW, kg, median (IQR)	9.7 (6.5‐15.0)	7.2 (4.3‐12.9)	.32
Sex, male, n (%)	9 (64.3)	5 (62.5)	1

Abbreviations: BW, body weight; CombPH, combined pre‐ and postcapillary pulmonary hypertension; IQR, interquartile range; IsoPH, isolated postcapillary pulmonary hypertension; PostPH, postcapillary pulmonary hypertension; PrePH, precapillary pulmonary hypertension.

**TABLE 3:** Pulmonary hypertension probability, underlying diseases, reported clinical signs, radiographic evidence of pulmonary edema, clinical evidence of right heart failure, and cardiovascular medications that the dogs were receiving at the time of inclusion in the study.

			PostPH (n = 22)	
		PrePH (n = 24)	CombPH (n = 14)	IsoPH (n = 8)
PH probability	Intermediate, n (%)	11 (46)^a^	0 (0)	7 (32)^b^
	High, n (%)	13 (54)^a^	14 (64)^b^	1 (4.5)^b^
Underlying diseases	Mild MMVD, stage B1, n (%)	13 (54)^a^	0 (0)	0 (0)
	MMVD, stage B2, n (%)	0 (0)	3 (14)^b^	2 (9)^b^
	MMVD, stage C, n (%)	0 (0)	10 (45)^b^	6 (27)^b^
	DCM, n (%)	0 (0)	1 (4.5)^b^	0 (0)
	HWD, n (%)	7 (29)^a^	0 (0)	0 (0)
	Chronic OAD, n (%)	4 (17)^a^	0 (0)	0 (0)
	Pneumonia, n (%)	2 (8)^a^	0 (0)	0 (0)
	Thoracic neoplasia, n (%)	2 (8)^a^	0 (0)	0 (0)
	Suspected PT, PTE n (%)	4 (17)^a^	0 (0)	0 (0)
	Suspected CIPF, n (%)	1 (4)^a^	0 (0)	0 (0)
	Not found, n (%)	4 (17)^a^	0 (0)	0 (0)
Clinical signs	Cough, n (%)	15 (62.5)^a^	10 (45)^b^	4 (18)^b^
	Respiratory effort, n (%)	11 (46)^a^	6 (27)^b^	2 (9)^b^
	Weight loss, n (%)	6 (25)^a^	8 (36)^b^	3 (14)^b^
	Lethargy, n (%)	9 (37.5)^a^	5 (23)^b^	1 (4)^b^
	Abdominal distention, n (%)	4 (17)^a^	3 (14)^b^	1 (4.5)^b^
	Syncope, n (%)	2 (8)^a^	4 (18)^b^	0 (0)
Radiographic evidence of pulmonary edema, n (%)		0 (0)	2 (9)^b^	1 (4.5)^b^
Clinical evidence of RHF, n (%)		3 (12.5)^a^	2 (9)^b^	0 (0)
CV therapy, n (%)		9 (37.5)^a^	10 (45)^b^	5 (23)^b^
Medications	P, ACEi, and F, n (%)	0 (0)	5 (23)^b^	3 (14)^b^
	P, S and F, n (%)	1 (4)^a^	1 (4.5)^b^	0 (0)
	P and F, n (%)	0 (0)	2 (9)^b^	2 (9)^b^
	S and F, n (%)	1 (4)^a^	0 (0)	0 (0)
	P, n (%)	0 (0)	2 (9)^b^	0 (0)
	F, n (%)	3 (12.5)^a^	0 (0)	0 (0)
	ACEi, n (%)	2 (8)^a^	0 (0)	0 (0)
	S, n (%)	1 (4)^a^	0 (0)	0 (0)
	P and ACEi, n (%)	1 (4)^a^	0 (0)	0 (0)
	P, ACEi, and F, n (%)	0 (0)	5 (23)^b^	3 (14)^b^

Abbreviations: ACEi, angiotensin converting enzyme inhibitors; CIPF, canine idiopathic pulmonary fibrosis; CombPH, combined pre‐ and postcapillary pulmonary hypertension; CV, cardiovascular; DCM, dilated cardiomyopathy; F. furosemide; HWD, heartworm disease; IsoPH, isolated postcapillary pulmonary hypertension; MMVD, myxomatous mitral valve disease; OAD, obstructive airway disorders; P, pimobendan; PH, pulmonary hypertension; PostPH, postcapillary pulmonary hypertension; PrePH, precapillary pulmonary hypertension; PT, pulmonary thrombosis; PTE, pulmonary thromboembolism; RHF, right‐sided congestive heart failure; S, sildenafil.

^a^ Percentage calculated for all dogs included in the PrePH group (n = 24).

^b^ Percentages calculated for all dogs included in the PostPH group (n = 22).

Precapillary PH secondary to pulmonary artery thromboembolism/thrombosis was suspected in 4 dogs (17%) affected by hyperadrenocorticism (n = 2), ehrlichiosis with secondary immune‐mediated hemolytic anemia (n = 1), and leishmaniasis (n = 1) with severe protein‐losing nephropathy. Idiopathic pulmonary fibrosis was suspected in 1 WHWT (4%) but was not confirmed. In 4 dogs (17%) in the PrePH group, no underlying cause of PH was found. Mild MMVD, classified as ACVIM stage B1, was present in 13 (54%) PrePH dogs (Table 3).

The underlying diseases diagnosed in the PostPH group were MMVD, ACVIM class C in 16 dogs (72%); MMVD, ACVIM class B2 in 5 subjects (23%); and dilated cardiomyopathy in 1 Great Dane (4%). Three dogs in the PostPH group (14%) showed radiographic evidence of pulmonary edema (Table 3).

**FIGURE 1:**
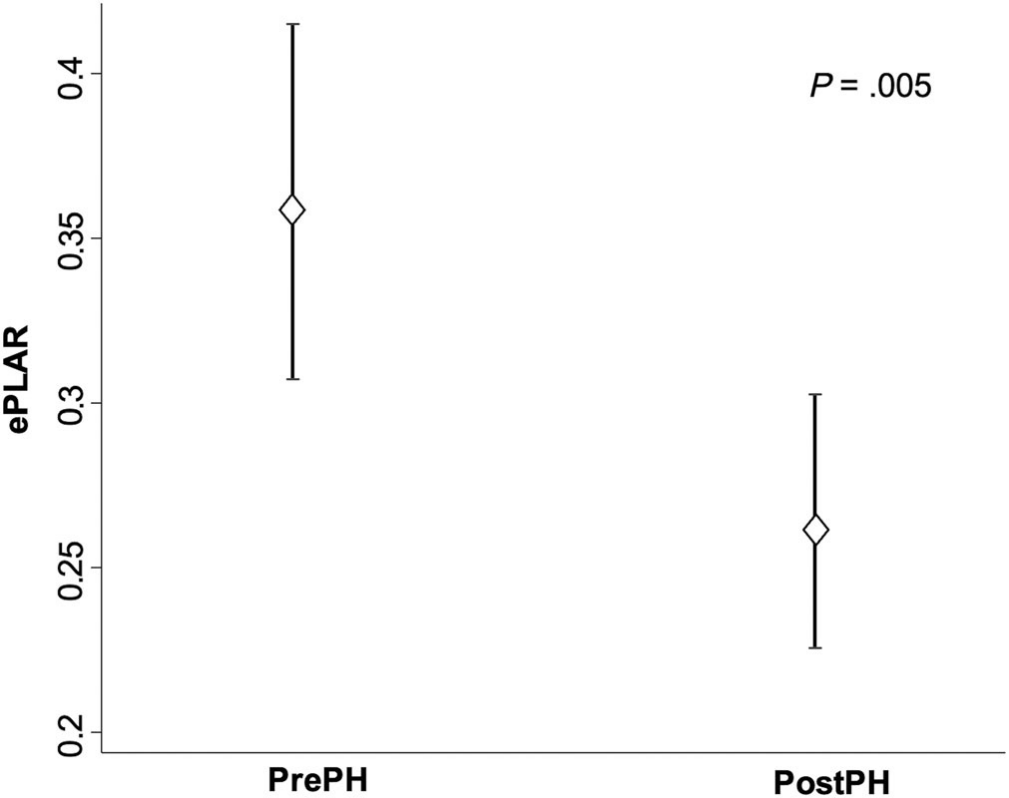
Graph showing the means and 95% confidence intervals ePLAR values of PrePH (n = 24) and PostPH (n = 22) groups. ePLAR, echocardiographic pulmonary to left atrial ratio index; PostPH, postcapillary pulmonary hypertension group; PrePH, precapillary pulmonary hypertension group.

**FIGURE 2:**
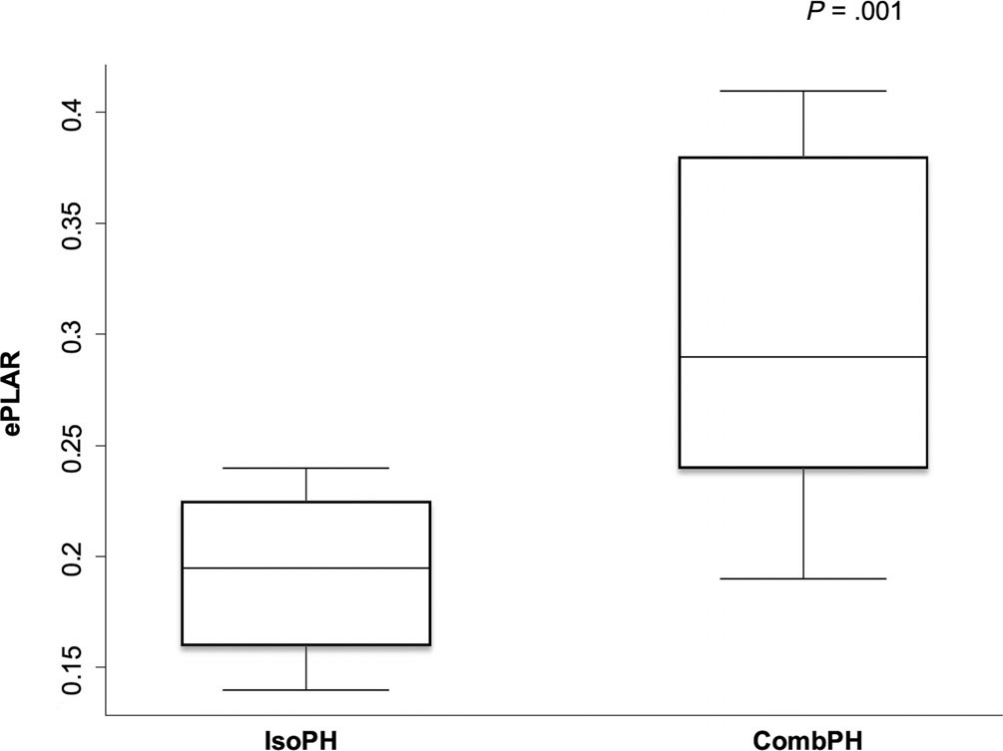
Boxplot graph showing the medians and interquartile ranges ePLAR values of IsoPH (n = 8) and CombPH (n = 14) subgroups. CombPH, combined pulmonary hypertension subgroup; ePLAR, echocardiographic pulmonary to left atrial ratio index; IsoPH, isolated pulmonary hypertension subgroup.

Comparisons of heart rate (HR) and echocardiographic variables between the Pre‐ and PostPH groups are shown in Table 4. The ePLAR index of the PrePH group was significantly higher compared with PostPH group [mean (SD) 0.36 (0.13) vs 0.26 (0.09), respectively; *P* = .005], as depicted in Figure 1. Comparisons of the HR and echocardiographic variables between the CombPH and IsoPH subgroups are shown in Table 5. The ePLAR index of the CombPH subgroup was significantly higher compared to that of the IsoPH group [median (IQR) 0.29 (0.24‐0.38), vs 0.20 (0.16‐0.23), respectively; *P* = .001], as depicted in Figure 2.

**TABLE 4:** Comparison of HR, and echocardiographic variables between Pre‐ and PostPH groups.

Variables	PrePH (n = 24)	PostPH (n = 22)	*P*‐value
HR, bpm, mean (SD)	116 (28)	129 (32)	.16
LA/Ao, mean (SD)	1.31 (0.2)	2.13 (0.36)	.00*
LVIDdN, median (IQR)	1.41 (1.24‐1.52)	2.14 (1.97‐2.28)	.00*
LVIDsN, mean (SD)	0.78 (0.21)	1.11 (0.4)	.00*
LV FS, %, mean (SD)	39 (10)	45 (13)	.11
LV EF, %, mean (SD)	62.3 (13.3)	68.2 (12.1)	.13
MMVD, n (%)	13 (54.2)	21 (95.5)	.00*
E, m/s, mean (SD)	0.65 (0.15)	1.27 (0.37)	.00*
*E*′ sept, m/s, mean (SD)	0.07 (0.02)	0.09 (0.03)	.00*
*E*/*E*′ sept, mean (SD)	9.44 (1.86)	14.19 (4.23)	.00*
TRV, m/s, mean (SD)	3.28 (0.89)	3.51 (0.75)	.35
ePLAR, m/s, mean (SD)	0.36 (0.13)	0.26 (0.09)	.00*
IVS flattening, n (%)	8 (33.3)	3 (13.6)	.12
RV hypertrophy, n (%)	15 (62.5)	6 (27.3)	.02*
RV dilation, n (%)	10 (41.7)	6 (27.3)	.31
TAPSE/Ao, mean (SD)	0.7 (0.16)	0.83 (0.16)	.06
RVOT FS, %, median (IQR)	46 (35.5‐51.5)	49 (44‐55)	.21
RV FAC, %, median (IQR)	45 (40‐49)	51.5 (43‐54)	.11
RV signs of PH, n (%)	22 (91.7)	14 (63.36)	.02*
PA/Ao, mean (SD)	0.98 (0.22)	1.05 (0.25)	.34
RPAD, %, mean (SD)	25.9 (12.2)	33.8 (13.1)	.07
AT, median (IQR)	53 (39‐82)	58.5 (34‐76)	.68
AT/ET, mean (SD)	0.33 (0.10)	0.35 (0.13)	.58
PF notching, n (%)	6 (25)	2 (9.1)	.25
PA signs of PH, n (%)	20 (83.3)	19 (86.4)	1
RA dilation, n (%)	11 (45.8)	2 (9.1)	.00*
CVC dilation, n (%)	7 (29.2)	3 (13.6)	.20
RA/CVC signs of PH, n (%)	11 (45.8)	3 (13.6)	.03*
PVRecho, median (IQR)	1.1 (0.6‐2.3)	1.6 (1.1‐2.7)	.07

Abbreviations: Ao, aortic diameter; AT, pulmonary flow acceleration time; AT/ET, pulmonary flow acceleration time to ejection time ratio; bpm, beats per minute; CombPH, combined pre‐ and postcapillary pulmonary hypertension; CV, cardiovascular; CVC, caudal vena cava; *E*, trans mitral pulsed wave Doppler‐derived early flow velocity; *E*/*E*′, quotient of the transmitral pulsed‐wave Doppler *E*‐wave velocity divided by the mitral annular septal Tissue Doppler Imaging *E*′‐wave; *E*′ sept, tissue Doppler imaging‐derived septal mitral annulus early velocity; ePLAR, echocardiographic pulmonary to left atrial ratio index; HR, heart rate; IQR, interquartile range; IsoPH, isolated postcapillary pulmonary hypertension; IVS, interventricular septum; LA, left atrium; LV EF, left ventricular ejection fraction obtained by biplane Simpson's method of disks; LV FS, left ventricular fractional shortening; LVIDdN, left ventricular internal diameter in diastole normalized to body weight; LVIDsN, left ventricular internal diameter in diastole normalized to body weight; MVD, mitral valve disease; PA, pulmonary artery; PA/Ao, main pulmonary artery to aortic diameter ratio; PF, pulsed wave‐derived pulmonary flow; PH, pulmonary hypertension; PostPH, postcapillary pulmonary hypertension; PrePH, precapillary pulmonary hypertension; PVRecho, echocardiographic‐derived pulmonary vascular resistance; RA, right atrium; RPAD, right pulmonary artery distensibility index; RV FAV, right ventricular fractional area change; RV, right ventricle; RVOT FS, right ventricular outflow tract fractional shortening; TAPSE/Ao, tricuspid annular plane systolic excursion normalized by aortic diameter; TRV, tricuspid regurgitation velocity.

* Significant *P* values (*P* < .05).

**TABLE 5:** Comparison of HR, and echocardiographic variables between CombPH and IsoPH subgroups.

Variables	CombPH (n = 14)	IsoPH (n = 8)	*P*‐value
HR, bpm, median (IQR)	141.5 (115‐160)	103.5 (87‐122.5)	.00*
LA/Ao, median (IQR)	2.2 (2‐2.4)	1.9 (1.8‐2.3)	.33
LVIDdN, median (IQR)	2.1 (2‐2.2)	2.2 (1.9‐2.3)	.56
LVIDsN, median (IQR)	1.1 (0.8‐1.3)	1.2 (0.7‐1.4)	.69
LV FS, %, median (IQR)	42 (39‐52)	44 (36‐55.5)	.99
LV EF, %, median (IQR)	73.5 (70‐85)	75 (67.5‐87)	.99
*E*, m/s, median (IQR)	1.3 (1‐1.7)	1.1 (1.1‐1.4)	.38
*E*′ sept, median (IQR)	0.1 (0.08‐0.1)	0.08 (0.06‐0.11)	.28
*E*/*E*′ sept, median (IQR)	13.1 (9.8‐17.1)	12.9 (11.8‐18)	.67
TRV, m/s, median (IQR)	4 (3.6‐4.2)	3.1 (2.3‐3.2)	.00*
ePLAR, m/s, median (IQR)	0.29 (0.24‐0.38)	0.20 (0.16‐0.23)	.00*
IVS flattening, n (%)	3 (21.4)	0 (0.0)	.27
RV hypertrophy, n (%)	5 (35.7)	1 (12.5)	.35
RV dilation, n (%)	5 (35.7)	1 (12.5)	.35
TAPSE/Ao, median (IQR)	0.9 (0.8‐0.9)	0.9 (0.6‐1)	.96
RVOT FS, %, median (IQR)	48 (46‐52)	51.5 (43‐56)	.61
RV FAC, %, median (IQR)	49.5 (42.5‐53.5)	53.5 (51‐58)	.16
RV signs of PH, n (%)	9 (64.3)	5 (62.5)	1
PA/Ao, median (IQR)	1.1 (0.9‐1.2)	0.9 (0.8‐0.1)	.04*
RPAD, %, median (IQR)	31.5 (14‐41)	40 (40‐42)	.2
AT, median (IQR)	45.2 (30‐64)	65 (53.5‐76.5)	.17
AT/ET, median (IQR)	0.3 (0.2‐0.5)	0.4 (0.3‐0.5)	.47
PF notching, n (%)	2 (14.3)	0 (0)	.52
PA signs of PH, n (%)	14 (100)	5 (62.5)	.04*
RA dilation, n (%)	2 (14.3)	0 (0)	.51
CVC dilation, n (%)	3 (21.4)	0 (0)	.27
RA/CVC signs of PH, n (%)	3 (21.4)	0 (0)	.27
PVR_echo, median (IQR)	2.3 (1.5‐3.4)	0.9 (0.4‐1.2)	.00*

Abbreviations: Ao, aortic diameter; AT, pulmonary flow acceleration time; AT/ET, pulmonary flow acceleration time to ejection time ratio; bpm, beats per minute; CombPH, combined pre‐ and postcapillary pulmonary hypertension; CV, cardiovascular; CVC, caudal vena cava; *E*, trans mitral pulsed wave Doppler‐derived early flow velocity; *E*/*E*′, quotient of the transmitral pulsed‐wave Doppler E‐wave velocity divided by the mitral annular septal Tissue Doppler Imaging *E*′‐wave; *E*′ sept, tissue Doppler imaging‐derived septal mitral annulus early velocity; ePLAR, echocardiographic pulmonary to left atrial ratio index; HR, heart rate; IQR, interquartile range; IsoPH, isolated postcapillary pulmonary hypertension; IVS, interventricular septum; LA, left atrium; LV EF, left ventricular ejection fraction obtained by biplane Simpson's method of disks; LV FS, left ventricular fractional shortening; LVIDdN, left ventricular internal diameter in diastole normalized to body weight; LVIDsN, left ventricular internal diameter in diastole normalized to body weight; MVD, mitral valve disease; PA, pulmonary artery; PA/Ao, main pulmonary artery to aortic diameter ratio; PF, pulsed wave‐derived pulmonary flow; PH, pulmonary hypertension; PostPH, postcapillary pulmonary hypertension; PrePH, precapillary pulmonary hypertension; PVRecho, echocardiographic‐derived pulmonary vascular resistance; RA, right atrium; RPAD, right pulmonary artery distensibility index; RV FAV, right ventricular fractional area change; RV, right ventricle; RVOT FS, right ventricular outflow tract fractional shortening; TAPSE/Ao, tricuspid annular plane systolic excursion normalized by aortic diameter; TRV, tricuspid regurgitation velocity.

* Significant *P* values (*P* < .05).

Using the Youden index, the best cutoff value of the ePLAR index to identify IsoPH was <0.245 [AUC at the cutoff point = 0.86, sensitivity (95% CI) = 0.71 (0.47‐0.95); and specificity (95% CI) = 1 (0.76‐1)]. Because the AUC obtained for ePLAR in distinguishing between the PrePH and PostPH groups was low (0.27), we did not evaluate an optimal cutoff to differentiate them.

Reliability test‐retest analysis showed a high degree of reliability for both intra‐ and interoperator measurements (ICC = 0.97, *P* < .0001; ICC = 0.94, *P* < .0001; respectively).

## 4 DISCUSSION

The results of the present study further support the hypothesis that ePLAR can be considered a valid noninvasive variable for hemodynamically classifying PH in dogs. Our findings demonstrate that ePLAR can differentiate IsoPH from CombPH, which is fundamentally important for optimizing the therapeutic management of PostPH in dogs.

**FIGURE 3:**
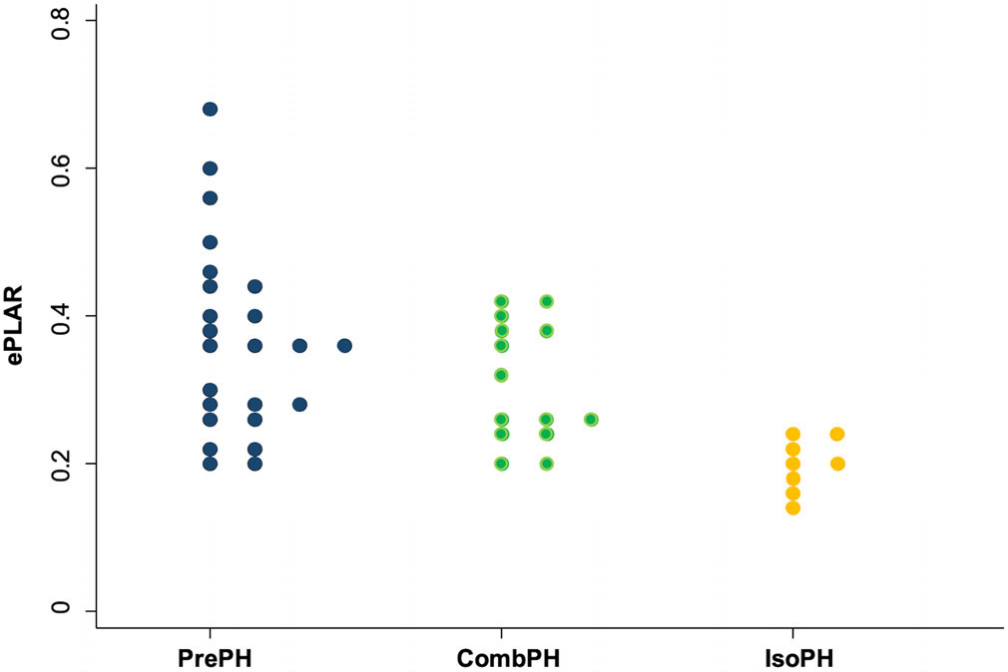
Dotplot graph showing ePLAR values of PrePH group (n = 24), CombPH subgroup (n = 14), and IsoPH subgroup (n = 8). CombPH, combined pulmonary hypertension subgroup; ePLAR, echocardiographic pulmonary to left atrial ratio index; IsoPH, isolated pulmonary hypertension subgroup; PrePH, precapillary pulmonary hypertension group.

The ePLAR index was obtained from the ratio between the Doppler‐derived TRV and the *E*/*E*′ ratio. In the absence of RV outflow tract obstruction, TRV represents an estimate of the systolic PAP, calculated by the simplified Bernoulli equation,^1^, ^2^ whereas the *E*/*E*′ ratio represents an index of LAP and PCWP, both in dogs and humans.^41^, ^42^, ^63^, ^64^ The ePLAR index has been validated against RHC in humans with PH.^39^ It can be considered an echocardiographic surrogate of the transpulmonary pressure gradient (TPG), which is the difference between PAP and PCWP, obtained by RHC.^39^, ^65^ In fact, both TPG and ePLAR follows the same trend, presenting higher values in subjects affected by PrePH and lower in those with PostPH.^39^ Our results confirm this thesis, indeed, they showed that dogs with PrePH had higher ePLAR values than those with PostPH. This finding is consistent with those previously reported in humans and dogs,^38^, ^39^ and it is physiologically plausible, considering that elevated TPG occurs during PrePH. However, despite this difference, it was not possible to establish a cutoff value for ePLAR to distinguish between the PrePH and PostPH groups. One possible explanation for this inconsistency could be that the CombPH subgroup dogs, in greater number than the IsoPH subgroup, with ePLAR values closer to those of the PrePH group (Figure 3), may have reduced the difference between the PrePH and PostPH groups.

The demographic characteristics of the dogs in the PrePH and PostPH groups showed no significant differences, except for BW, which was significantly higher in the PrePH dogs. The reason for this difference could be that dogs with PostPH were more affected by MMVD, which is more common in small dogs.^66^ As previously reported,^2^, ^13^ PrePH dogs had reduced LV internal diameters (LVIDdN and LVIDsN) compared with PostPH dogs as a consequence of (1) reduced venous return to the left heart in PrePH dogs and (2) LV remodeling secondary to LHD in PostPH subjects.^2^ Our results showed that the echocardiographic signs of PH affecting the right heart, such as RV hypertrophy and RA dilatation, were significantly more frequent in dogs with PrePH. This implied a clinically relevant difference in the severity of PH between the 2 groups. These findings are similar to those previously reported in humans and dogs.^38^, ^67^ As we did not find any significant differences in TRV between PrePH and PostPH, the cause of this result is difficult to discern. Although TRV does not accurately estimate PAP,^68^ its value increases with increasing PVR and PH severity.^34^, ^61^ However, right heart adaptation and remodeling in PH is a complex process that depends not only on the severity of pulmonary vascular disease but also on the interaction between neurohormonal activation, coronary perfusion, myocardial metabolism, rate and time of onset of PH, its underlying etiology, and genetic factors.^69^

Another important finding in our study was that the ePLAR index was significantly higher in dogs with CombPH than in those with IsoPH. This result further supports the association between ePLAR and TPG and is consistent with previous observations reported in humans.^39^ Postcapillary PH is a common cause of PH in dogs, which can occur because of any type of LHD, but it is more prevalent in dogs with MMVD and myocardial diseases.^24^ Postcapillary PH begins primarily because of the passive transmission of elevated left heart filling pressures into the pulmonary veins and capillaries. In this initial and reversible stage (IsoPH), the RV generates higher systolic pressure to overcome pulmonary venous hypertension and ensure an adequate transpulmonary pressure gradient. However, chronic pulmonary venous hypertension and hypoxia determine pulmonary arterial vasoconstriction and remodeling, which leads to an irreversible increase in PVR and, therefore, CombPH.^70^

In this study, we identified an optimal ePLAR cutoff value of 0.245 to differentiate between IsoPH and CombPH. The differences between IsoPH and CombPH may have important implications for the therapeutic management of dogs with PostPH. First, administration of PDE5i to dogs with IsoPH could potentially precipitate or worsen pulmonary edema. A pulmonary artery vasodilator could cause a sudden increase in pulmonary perfusion, and therefore, in pulmonary venous return to the LA, thereby triggering or worsening the pulmonary edema.^2^, ^36^ Second, a lack of benefit in using PDE5i in dogs with an IsoPH phenotype can be found, as described in human medicine.^37^ In veterinary medicine, there are not enough data; however, ACVIM guidelines recommend cautious use of PDE5i in patients with PH secondary to LHD.^2^

Not surprisingly, all dogs with CombPH showed echocardiographic evidence of pulmonary artery anatomical abnormalities, such as increased main PA diameter, which is correlated with RHC‐derived PAP and PVR.^71^, ^72^

As reported in Tables 4 and 5, the quantitative variables other than ePLAR showed significant differences between the study groups. In the comparison between PrePH and PostPH, the LA/Ao, transmitral *E*‐wave velocity, and *E*/*E*′ ratio showed significant differences. Similarly, ePLAR, TRV, and PVRecho showed significant differences between CombPH and IsoPH. These differences can be explained in part by the strong correlation between some of these variables and the ePLAR index (eg, TRV and *E*/*E*′ constitute the ePLAR numerator and denominator, respectively). However, other variables, such as LA/Ao and PVRecho, differed because of the classification criteria used. Further studies, in which RHC is used as the gold standard method of diagnosis, are needed to verify whether these differences persist and which of these echocardiographic variables has the best diagnostic accuracy in the hemodynamic classification of PH in dogs. However, in dogs with PH and concurrent respiratory and hemodynamically significant LHD, the ePLAR index could be more accurate than its individual components in distinguishing which hemodynamic form prevails, and therefore, in helping clinicians in the therapeutic management and monitoring of patients.

The current study found that the ePLAR had a high degree of intra‐ and interoperator measurement repeatability. A possible explanation for this result is that ePLAR was obtained from the measurement of 3 Doppler variables (*E*, *E*′, and TR velocities) easily obtainable and measurable by expert operators.

The lack of a definitive diagnosis and classification of PH by RHC were the most notable limitations of this study. Both PH diagnosis and classification were based on echocardiographic variables, which may have influenced the ability of ePLAR to differentiate between PH pathophysiology.

For example, echocardiography‐derived LA size was used to distinguish between Pre‐ and PostPH. This approach assumes that all dogs with LA enlargement have elevated LAP and that all dogs with normal LA size have normal LAP, which is not always true. Furthermore, we used the echocardiography‐derived PH probability and PVRecho to distinguish CombPH from IsoPH. In humans, PVRecho has been validated against RHC,^73^, ^74^ and in dogs with LHD it has been demonstrated to increase as the probability and severity of PH increases.^61^ However, cutoff values for PH probability and PVRecho for differentiating CombPH from IsoPH in dogs have not been established. Therefore, our choice to consider dogs with PostPH with a high probability of PH, associated with PVRecho ≥1.11, as affected by CombPH, could have led us to misclassify some patients into the 2 PostPH subgroups.

Dogs with concurrent respiratory and hemodynamically significant LHD were excluded based exclusively on anamnestic, clinical, and radiological results. Not all dogs in the PostPH group underwent arterial blood gas analysis; therefore, some subclinical respiratory diseases associated with hypoxia and/or vascular remodeling with increased PVR could have affected the dogs at the time of inclusion in the study. This may have resulted in the inclusion of dogs with mixed forms of PH (PrePH and PostPH) and increased PVRecho in the CombPH subgroup. Furthermore, 2 dogs classified as having CombPH presented radiographic evidence of cardiogenic pulmonary edema; however, we do not know whether the increase in PVRecho was due to transitory hypoxia‐induced vasoconstriction or vascular remodeling secondary to chronic LHD. Another source of error in classification could be the fact that in some dogs, it was not possible to achieve a definitive diagnosis of the causative disease (s) of PH. Although ePLAR was significantly different between the Pre‐ and PostPH groups, these possible misclassifications could explain the extent of the gray zone among the PH pathophysiology groups (Figure 3).

Another source of uncertainty is that a high percentage of dogs (52%) were receiving cardiovascular medications (s) at the time of inclusion in the study. Cardiovascular drugs may affect pulmonary pressure and resistance; therefore, some dogs may have been misclassified. Another limitation was the small number of recruited subjects with a small sample size; therefore, caution must be exercised in generalizing the results. Finally, it is important to remember the main technical limitation of the ePLAR index, which is the possibility of being calculated only in subjects with TR, excluding cases of PH in which this is absent.

In conclusion, our study showed that the ePLAR index can be considered a valid noninvasive variable for hemodynamically classifying PH in dogs. Correct differential diagnosis allows for a deeper understanding of the physiopathology of the condition and can have a significant impact on patient management. Further studies with larger sample sizes and patient classifications based on RHC are required to better understand the clinical utility of the ePLAR index.

## CONFLICT OF INTEREST DECLARATION

Authors declare no conflict of interest.

## OFF‐LABEL ANTIMICROBIAL DECLARATION

Authors declare no off‐label use of antimicrobials.

## INSTITUTIONAL ANIMAL CARE AND USE COMMITTEE (IACUC) OR OTHER APPROVAL DECLARATION

The study protocol was approved by the Ethical Committee for Animal Welfare of the University of Sassari (OPBSA), protocol number 32346 of 18 March 2022.

## HUMAN ETHICS APPROVAL DECLARATION

Authors declare human ethics approval was not needed for this study.
